# Insights Into Parkin-Mediated Mitophagy in Alzheimer's Disease: A Systematic Review

**DOI:** 10.3389/fnagi.2021.674071

**Published:** 2021-07-29

**Authors:** Sepideh Goudarzi, Asieh Hosseini, Mohammad Abdollahi, Hamed Haghi-Aminjan

**Affiliations:** ^1^Department of Toxicology and Pharmacology, Faculty of Pharmacy, Tehran University of Medical Sciences, Tehran, Iran; ^2^Razi Drug Research Center, Iran University of Medical Sciences, Tehran, Iran; ^3^Department of Toxicology and Pharmacology, School of Pharmacy, and Toxicology and Diseases Group, Pharmaceutical Sciences Research Center, The Institute of Pharmaceutical Sciences, Tehran University of Medical Sciences, Tehran, Iran; ^4^Pharmaceutical Sciences Research Center, Ardabil University of Medical Sciences, Ardabil, Iran

**Keywords:** Alzheimer's disease, amyloid–beta, mitophagy, PINK 1, Parkin (PARK2)

## Abstract

**Background:** Parkin-mediated mitophagy is the dominant mitophagy pathway of neural cells. Its restoration will result in prevention of cognitive decline, including Alzheimer's disease (AD). The role of this mitophagy pathway in neurodegenerative diseases has drawn attention in recent years. The two main pathological proteins in AD, amyloid β (Aβ) and human Tau (hTau), interfere with mitochondrial dynamics through several pathways. However, taking into consideration the specific interactions between Aβ/hTau and Parkin, special focus is required on this mitophagy pathway and AD. In this review, these interactions are fully discussed, and an overview of the neuroprotective drugs that enhance Parkin-mediated mitophagy is presented.

**Methods:** This systematic review was performed according to PRISMA guidelines, and a comprehensive literature search was done in the electronic databases up to September 2020, using search terms in the titles and abstracts to identify relevant studies. One hundred eighty-six articles were found, and 113 articles were screened by title and abstract. Finally, 25 articles were included in this systematic review according to our inclusion and exclusion criteria.

**Results:** Accumulation of Aβ and hTau affects mitophagy, including Parkin-mediated. Tau seems to prevent Parkin translocation directly. A Parkin level in the cell appears to be of importance in determining the damage caused by Aβ and hTau and in the future therapeutic approaches. Parkin controls the PINK1 level *via* the presenillins, suggesting that mutations in presenillins affect Parkin mitophagy.

**Significance:** Parkin mitophagy is a process affected by several AD pathological events multidimensionally.

## Graphical Abstract

Interactions of PINK1/Parkin pathway with AD-related proteins. 
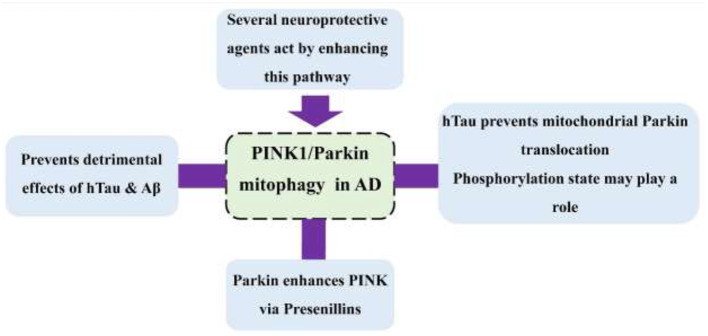

## Introduction

Parkin (also known as “ubiquitin E3 ligase”) is a molecule of high interest in the recent decade due to its several fundamental roles in the cells, including mitochondrial homeostasis maintenance. It was first found as one of the proteins involved in the pathogenesis of Parkinson's disease, as its loss causes neuro-inflammation and degeneration. It is then not surprising that literature also indicates that this protein has a dominant role in reversing the cognition deficits and preventing Alzheimer's disease (AD) (Martín-Maestro et al., 2016; Fang et al., 2019). It appears that this protein is accumulated where most involved with AD pathology (hippocampus) and seems to be of great importance in the future AD treatments (Gao et al., 2020). During recent years, however, mitophagy on Parkinson's disease has been massively studied.

It is known that mitophagy effectively attenuates cognitive decline in AD (Fang et al., 2019). Although there is basal mitophagy activity as a normal function (McWilliams et al., 2018), this action becomes of great importance for stressed mitochondria. Upon mitochondrial stress outriding the anti-inflammatory defense system, mitochondria release further oxidative species, causing a vicious circle ending in a dysfunctional mitochondrion. Enough number of these waste bodies will result in cellular apoptosis, following their release of apoptotic factors, such as the cytochrome C. The key protection of the neuron in this condition is mitophagy, which corresponds to the removal of the dysfunctional mitochondria before the neuron is down to apoptosis. Although mitophagy pathways are not frequent, studies on enhancement of Parkin-mediated mitophagy have reported promising outcomes. This implies the Parkin-mediated pathway to be the dominant way of mitophagy under stress. Moreover, it is still not clear what the mechanisms of the others, including their induction pathways, would be (Villa et al., 2018).

Moreover, reviews of the role of this pathway in AD have not addressed the mechanisms of the specific proteins of Alzheimer's and the Parkin mitophagy. Additionally, there seems to be a lack of information in this specific area, while there is diffuse information on these pathways that urges us to bring them together to conclude how they influence each other. In this systematic review, characteristics of Parkin-mediated mitophagy in AD and the role of amyloid β (Aβ) and hTau and their related proteins in this pathway are discussed. Finally, potential enhancers of the pathway are reviewed.

## Methodology

### Study Protocol

The present systematic review study was performed based on a previous study (Moher et al., 2009). A comprehensive systematic search of the following electronic databases: PubMed, Embase, ProQuest, Scopus, and Web of Sciences according to the study search terms, which are based on the aims of the present study, including “Parkin RBR E3 ubiquitin protein ligase” or “PRKN” or “PDJ” or “AR-JP” or “LPRS2” or “PARK2” or “Park” or “Pdr-1” or “parkin” “PARK 2” and “mitochondrial degradation” or “mitophagy” and “Alzheimer's disease” or “Alzheimer” or “Alzheimer's disease” or “senile dementia” or “primary senile degenerative dementia” or “acute confusional senile dementia” or “senile dementia, acute confusional” or “dementia, presenile” or “presenile dementia,” was done up to September 27, 2020.

### Eligibility Criteria

After entering all the manuscripts searched from five databases into a reference, all the studies were initially screened based on the study aims in the title and abstract, including [1] all eligible articles that studied specific characteristics of Parkin-mediated mitophagy in AD (i.e., AD models) or studies on specific interactions between Aβ/hTau and Parkin [2], the studies with comprehensive and sufficient information, [3] the studies without limitation in *in vitro* and *in vivo*, and [4] the studies without restriction on the publication year. For exclusion criteria, we excluded [1] letters, [2] posters, [3] articles in languages else than English, [4] and studies on Alzheimer's disease and mitophagy, in which no conclusions were obtained exclusively on the Parkin pathway, e.g., mitochondrial dysfunction and AD.

### Data Collection

After deleting duplicate articles by two reviewers (SG and HHA), the articles were screened independently based on the previously expressed keywords. The full texts of the articles were separately evaluated, and the related articles were entered based on the inclusion and exclusion of the relevant predefined criteria. Those inconsistent with the objectives and predefined criteria of the present study were removed upon agreement of the two reviewers.

## Results

### Literature Search and Screening

**Figure 1:**
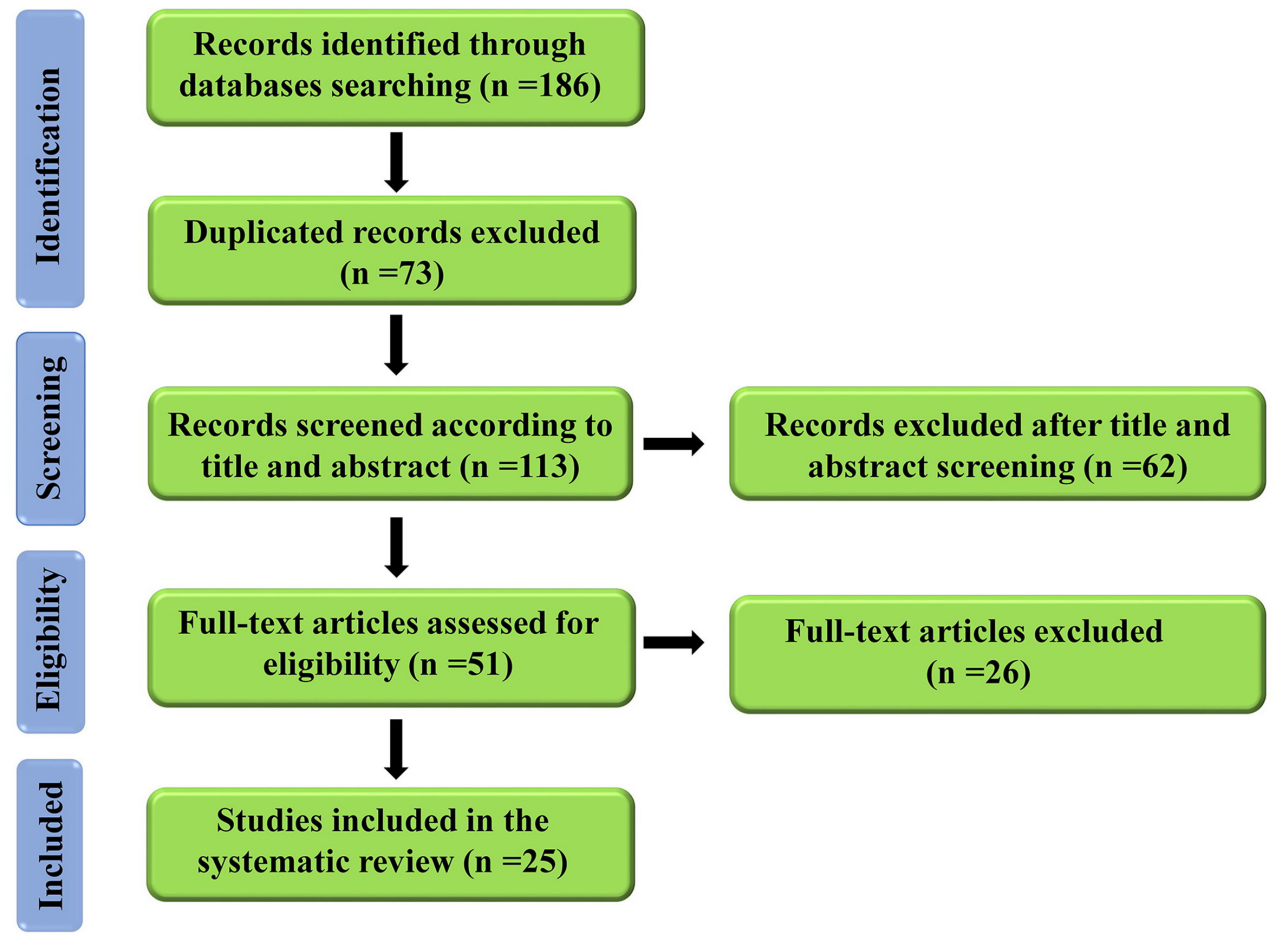
A flow diagram of the selection process for the present study.

Based on our systematic search keywords, one hundred and eighty-six articles were found in electronic databases. After deleting the duplicate articles, 113 articles were screened based on prespecified keywords in the title and the abstract, and 62 articles were removed. Then, 51 full-text articles were evaluated for the second screening based on the predefined criteria. Eventually, 25 articles qualified to enter our systematic review. The process of searching in electronic databases and its two-step screening are shown in Figure 1.

### Data Extraction

The data of each article were extracted by the author SG and compiled in Table 1, and rechecked by the author HHA under the following titles: (1) First author and year of publication, (2) models (*in vitro* or *in vivo*)/a route of administration, dosage, duration of treatment (3) *in vivo* assessments (4) the Parkin level in the AD model vs. control, (5) the Parkin level in the treated group vs. the AD model, (6) other related markers.

**Table 1:** Studies on Parkin level alterations.

**Data**								
**References**	**Model (** ***in-vivo/in-vitro*** **)**	**Intervention (dosage)/Route of administration/ Treatment time**	**Autophagy dysfunction**	***In-vivo*** **assessments**	**Parkin assessment**	**Parkin level in AD vs. normal**	**Parkin level in treated group vs. AD**	**Other parkin- related markers**
Zhao et al. (2020)	APP/PS1 mice	Treadmill exercise/5 days of week, for 12 weeks	-	MWM	WB (of hippocampal mitochondria fractions)	Parkin sig. increased	Parkin sig. increased	LC3II, the same pattern as parkin
Gao et al. (2020)	APP/PS1 mice	-	-	-	IHC WB	Parkin sig. increased (esp. in CA1 and CA2)	-	PINK1 sig. increased (esp. in CA1 and CA2)
Fang et al. (2019)	APP/PS1 mice	Urolithin A, Actinonin/Oral/2 months	-	MWM, Y maze, Object recognition	WB	-improved mitophagy but not parkin in detail	UA increased parkin	PINK1 increased
	*C. elegans*	Urolithin A, Actinonin/-/-	-	Olfactory learning Chemotaxis assay	WB	-	-	-
Yang et al. (2020)	*C. elegans*	6”‘-Feruloylspinosin/-/36 h	-	Chemotaxis assay, Odorant preference assay	WB	-	-	-
Han et al. (2020)	IT Aβ injection	β-Asarone (15,30,45 mg/kg)/i.p./30 days	-	MWM	WB IHC	Parkin sig. decreased in AD models	Parkin sig. increased in treated, but not as much as normal	PINK the same as parkin
Sun et al. (2020)	APP/PS1	Melatonin (0.5 mg/day)/oral/4 months	-	MWM	WB	Parkin increased	Parkin sig. decreased in treated	PINK sig. decreased in treated
Wang et al. (2018)	PC12 cells	Resveratrol (1,3,10,30 μM)/-/24 h	Aβ	-	WB	Parkin sig. increased	Parkin sig. increased	LC3II and Beclin-1With the same pattern as Parkin
Gao et al. (2020)	BV2 cells	TSG(10 μM, 1 μM, 100 nM, and 10 nM)/-/24 h	LPS	-	WB IHC	Parkin sig. increased	Parkin sig. increased	PINK1the same pattern as Parkin
Fang et al. (2019)	Human neuronal SH-SY5Y cells	Urolithin A/Actinonin (ranging from 10 to100 μM)/-/-		-	WB	Not mentioned in the statistics	Not mentioned in statistics	PINK1, Beclin-1, Bcl2L13 increased via treatment
Yang et al. (2020)	PC12 cells	6”‘-Feruloylspinosin (50, 100 and 200 μM)/-/-	Aβ	-	WB	Parkin sig. decreased	Parkin sig. increased, but not as much as normal	PINK1 the same pattern as parkin
Xiong et al. (2020)	APP695swe cells	Valinomycin(1 μM)/-/3, 6, 12 h	APP	-	WB	Parkin sig. decreased	parkin insig. increased, Parkin recruitment to mitochondria sig. increased	PINK1 and LC3II sig. increased via treatment
Hirano et al. (2019)	Human neuronal SH-SY5Y cells from: control, PARK2 def., PARK6 def.	Memantin (100 mM)/-/24 h	PARK2 and PARK7 deficiency	-	Not assessed	Less than normal since it was PARK2 mutant	-only mitophagy was increased	LC3II sig. increased

*MWM, Morris Water Maze; WB, Western blot; sig., significantly; IHC; immunohistochemistry; IT, intrathecal; LPS, lipopolysaccharide; def., deficiency*.

### The Included Studies Could Be Categorized Into Two Areas

*Studies on how Amyloid* β *and hTau affect Parkin-mediated mitophagy and vice versa* (Michiorri et al., 2010; Khandelwal et al., 2011; Corsetti et al., 2015; Wang X. et al., 2015; Ye et al., 2015; Hu et al., 2016; Martín-Maestro et al., 2016, 2017, 2019; Checler et al., 2017; Goiran et al., 2018; Castellazzi et al., 2019; Cummins et al., 2019; Fang et al., 2019; Reddy and Oliver, 2019; Wang et al., 2020). The main results were the following:

It seems that the PINK/Parkin pathway has several roles in the prevention of AD. Generally, accumulation of Aβ and hTau affects mitophagy, but Tau directly prevents Parkin translocation; however, the mechanism is not yet ascertained. The Parkin level seems to be a potential indicator of mitochondrial damage and the rate of cognitive decline. Not only does PINK1 recruit Parkin to the mitochondria, but Parkin enhances the PINK1 level *via* presenilins, and, thus, mutations in presenilins impair the PINK/Parkin mitophagy cycle.

Studies that evaluated potential neuroprotective agents in AD models that ameliorate neurodegeneration *via* enhancement of Parkin-mediated mitophagy (Wang et al., 2018; Fang et al., 2019; Hirano et al., 2019; Gao et al., 2020; Han et al., 2020; Sun et al., 2020; Xiong et al., 2020; Yang et al., 2020; Zhao et al., 2020, 2021). The studies used both *in vivo* and *in vitro* models of AD, and the outcomes mainly included increased the Parkin level or related proteins. The main results were the following: (Table 1 provides the details.).

PINK1 apparently starts to increase upon mitophagy enhancement prior to Parkin, taken from the fact that studies, which measured both the Parkin and PINK1 relative levels, reported a higher increase in PINK1 (Gao et al., 2020; Han et al., 2020; Xiong et al., 2020). In higher doses or exposures, effectiveness of these compounds seems to decrease, indicated by the PINK1 and Parkin levels (Gao et al., 2020; Xiong et al., 2020).

## Discussion

This was the first study to bring together Parkin-mediated mitophagy characteristics focused on its interactions with proteins of AD. There seem to be complex interactions between these proteins, which indicate new roles contributing to pathology of AD, hence suggesting potential new targets for treatment.

### Parkin-Mediated Mitophagy and AD

PINK1 (PTEN-induced kinase 1) is the well-known pivotal protein in the Parkin pathway, induced by PTEN (phosphatase and tensin homolog). Upon mitochondrial damage, depolarization of mitochondrial membrane causes translocation of cytosolic Parkin to the membrane, as well as PINK1. PINK1 not only induces translocation of Parkin but also phosphorylates it and activates its ligation. Ubiquitination of the outer membrane mitochondria (OMM) proteins will induce pathways, leading to autophagy of the organelle. Moreover, PINK1 seems to have interactions in autophagy pathways upstream to the Parkin, including induction of the protein Beclin-1 (Michiorri et al., 2010). Beclin-1 is one of the proteins involved in autophagy and is required to induce it. It appears to play a role in the PINK/Parkin mitophagy pathway. In an *in vivo* model of AD, it is reported that intracellular Aβ accumulation induces a beclin-independent autophagy pathway, which leads to accumulation of the dysfunctional mitochondria. However, by ubiquitinating the intracellular Aβ, Parkin induces the beclin-dependent pathway and leads to removal of the vesicles, containing mitochondria debris. Ubiquitination of the Aβ will also prevent the extracellular plaque formation (Khandelwal et al., 2011).

#### Aβ and Tau Impair Parkin-Mediated Mitophagy

Aβ and Tau cause disruptions in mitophagy (Fang et al., 2019) through abnormal interactions with mitochondrial fission proteins and the Parkin (Reddy and Oliver, 2019). In the study by Wang et al. cells treated with Aβ had a greater number of mitochondria than the normal, indicating the inability to remove dysfunctional mitochondria. Moreover, the cells exhibited more of the dysfunctional and misshaped ones and autophagosomes containing mitochondria (Wang et al., 2020).

A study by Hu et al. indicated that human N-terminal Tau (but not C-terminal, phosphorylated tau) is found in the outer mitochondrial membrane. This causes an increase in its membrane potential and hyperpolarizes the membrane, preventing Parkin translocation (Hu et al., 2016). Furthermore, phosphorylation state of hTau directly influences its membrane localization, where phosphorylation and mutations in the N terminal limit transport of tau within neuronal membrane (Pooler et al., 2012). Investigating further roles of this protein in this pathway by Cumminis et al. it was found that human Tau (hTau) protein prevented Parkin translocation by another mechanism. The team showed that cytosolic Parkin was trapped by hTau, although this was only observed in cytosol and not in the mitochondrial membrane. However, the authors declare that they did not observe OMM hyperpolarization by Tau (Cummins et al., 2019), compared with the study of Hu, neither have they discussed the phosphorylation state.

**Figure 2:**
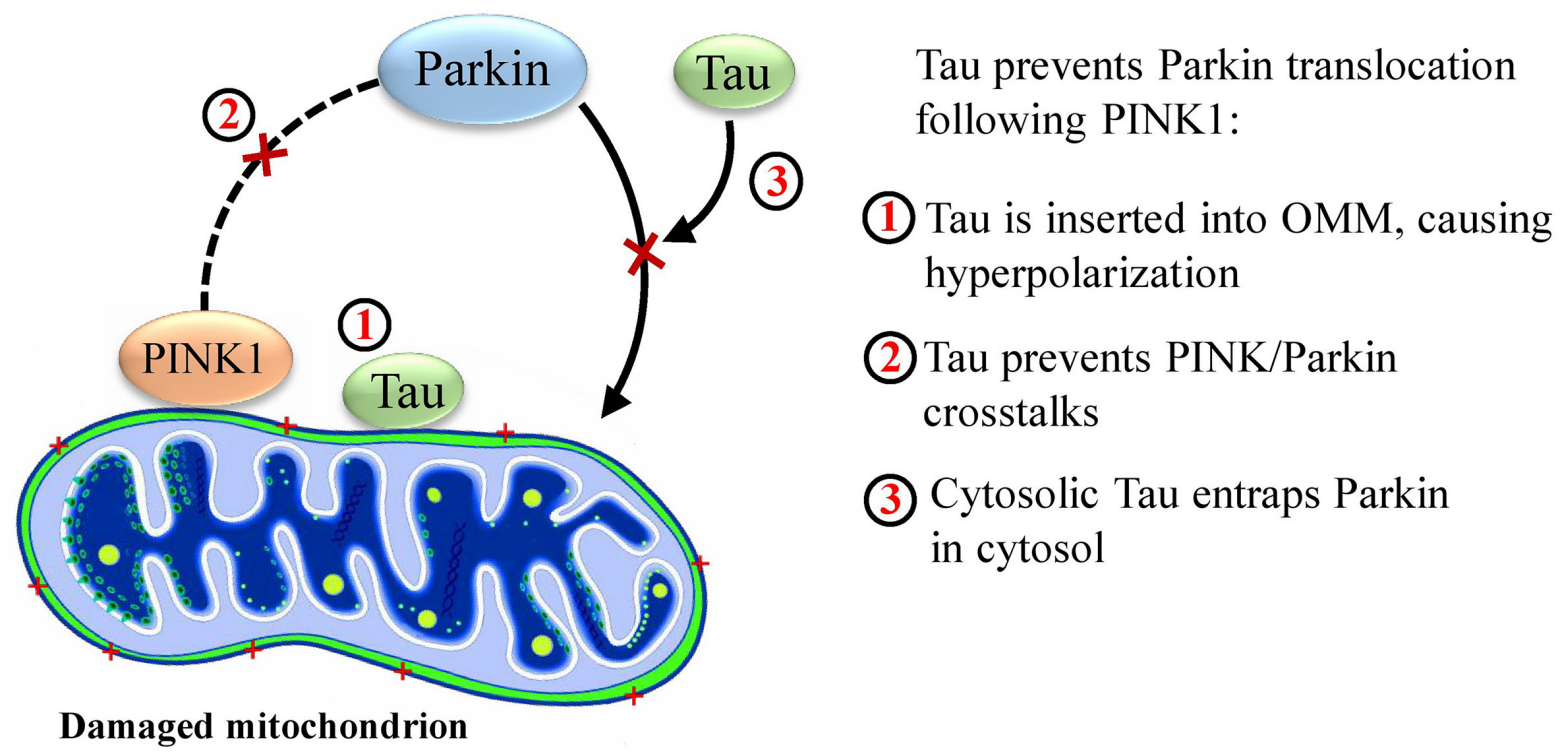
Probable mechanisms by which Tau disrupts mitophagy.

A recent *in vitro* study has pointed out the deteriorating synergistic effect of amyloid precursor protein (APP) and Tau on mitophagy. In their model, overexpression of Tau increased the basal Parkin level and prevented Parkin translocation, which was exacerbated *via* the presence of APP. The exciting finding was that overexpression of these two proteins did not interrupt proper stabilization of PINK1 in the mitochondrial membrane, while Parkin translocation was interrupted. This suggests the existence of unknown cross talks between PINK1 and Parkin, which is altered by the presence of APP and Tau (Martín-Maestro et al., 2016). This is in accordance with the findings of Hu et al. and Cumminis et al., in which Tau prevented Parkin translocation. Figure 2 depicts the probable disrupting mechanisms of Parkin translocation by Tau.

The effect of AD protein on mitophagy has also been approved clinically. In a recent study by Castellazzi et al., serum samples of patients at different stages of cognitive impairment due to amyloidopathies have had lower amounts of Parkin and Atg (autophagy-related proteins), being markers of autophagy and mitophagy. It was, therefore, suggested that these biomarkers could be of use in early diagnosis of the disease (Castellazzi et al., 2019).

Nevertheless, considering the alleviating mechanisms of mitophagy in neurodegeneration, it is not far from mind that mitophagy is a defense system of the cell against disruption of these proteins. An increase in Parkin, normally, is observed in the hippocampus of APP/PS1 transgenic mice, especially in the areas of CA1 and 2 (Wang X. et al., 2015). Wang et al. further explored alleviating the effects of Aβ on mitochondria through increasing the expression of Parkin. As expected, cells overexpressing Parkin treated with Aβ had better mitochondrial functions and less ROS production (Wang et al., 2020). On the other side, it is reported that an endogenous-truncated form of hTau (NH2-26-230 tau fragment) is found in synaptosomal mitochondria of AD patients much higher than in normal, and this causes excessive mitochondrial elimination due to over activation of the mitochondrial turnover, regulating systems by abundant Parkin recruitment (Corsetti et al., 2015). However, the authors do not discuss the phosphorylation state of this Tau form (which appears to be non-phosphorylated) in order for us to compare it with the previous statements on the effect of Tau on Parkin membrane localization, which were on the contrary to the present statement.

Up to this, it seems a circle has existed in which Aβ and Tau disrupt this mitophagy, and the mitophagy protects mitochondria from their actions. The fact is mitochondrial impairment and OMM depolarization that are caused by Aβ and hTau increase Parkin and Parkin translocation, whereas Aβ and hTau themselves prevent Parkin translocation and decrease Parkin. It appears that, in the first stages of pathology, the equation is, finally, in favor of increased Parkin, although not enough to defend mitochondrial homeostasis against AD pathological occurrences. However, in later stages, where abundance of Aβ and hTau exists; Parkin and the mitophagy mechanisms cannot overcome. In most of the molecular studies on Parkin in AD, it is mentioned that Parkin levels were significantly higher in AD mice compared with the healthy controls. In the study of Castellazzi et al., patients with MCI (mild cognitive impairment) have had higher maximum levels of Parkin even than the control of the same age. However, their median levels were as low as AD patients. This may be because of the gap between molecular and clinical observations, in which there might be substages to mild cognitive impairment as well (i.e., patients in later stages of MCI have had Parkin serum levels similar to patients with AD, whereas those in earlier stages had significantly higher).

Improved microglial activity and phagocytosis were also observed in an *in vivo* study on APP/PS1 transgenic mice, probably by enhancing energy production and transfer, following effective mitophagy (Fang et al., 2019).

#### The Role of Presenillins in Parkin-Mediated Mitophagy

**Figure 3:**
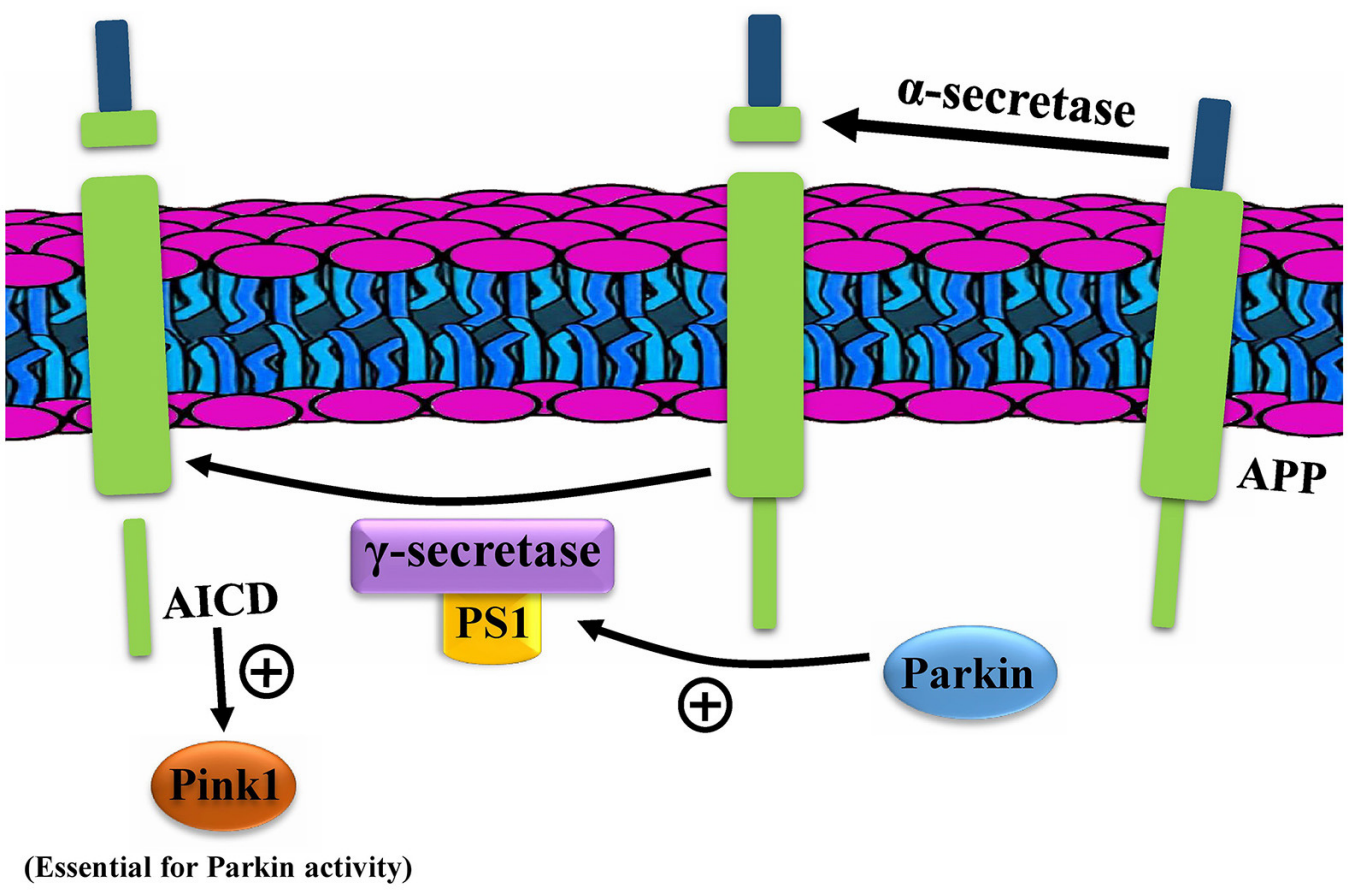
Parkin enhances PINK1 through presenillins (PS). AICD, APP intracellular domain; APP, amyloid precursor protein.

Whether the APP is cleaved primarily by alpha or beta-secretase, it is then cleaved by gamma-secretase, whose active site is mainly constituted of Presenillins 1 and 2. These two proteins are shown to modulate PINK1 expression, independent of PTEN (Checler et al., 2017). Although PS1 may have several roles in the cell, it appears that its effect on PINK is dependent on its gamma-secretase activity since the effects were disrupted by inhibitors of this enzyme (Checler et al., 2017). Explorations revealed that the APP intracellular domain (AICD), a product of cleavage by PS1 in gamma-secretase, along with FOXO3, increases PINK1 promoter transactivation, a mRNA level, and protein expression (Checler et al., 2017; Goiran et al., 2018). This is while PS2 decreases the PINK1 level. What is surprising is that PARK2 modulates both PS1 and PS2 in a manner opposite of their effects on PINK1. This provides support for the hypothesis that PARK2 increases the PINK1 level *via* both the PS1 and PS2. It has been generally thought that PINK1 is an upstream protein of Parkin in its mitophagy pathway; while this finding proves that Parkin (and the gene PARK2) could modulate the levels of PINK1 upstream to the mitophagy, causing a cycle in which each enhances the other in the process of mitophagy (Figure 3).

Mutations in these proteins, especially PS1, are the most frequent cause of familial AD due to unfancied cleavages, producing harmful Aβ peptides. Added to that are the disrupted PINK-Parkin cycle and mitophagy (Martín-Maestro et al., 2017).

### Enhancing Parkin-Mediated Mitophagy Against AD

As mitophagy has been found to be a promising factor in alleviating cognitive decline, recent studies have assessed the effects of neuroprotective compounds and exercise on mitophagy, and, dominantly, Parkin-mediated mitophagy.

#### Exercise

Twelve weeks of treadmill exercise for APP/PS1 mice upregulated Parkin, enhanced mitophagy, and decreased Aβ levels (Zhao et al., 2020). However, PINK1 alteration patterns were not consistent with the rest (Zhao et al., 2020). NAD-dependent deacetylase sirtuin-1 (SIRT1) is an interesting protein with several protective roles in the body. It plays a role in alleviating inflammaging (chronic inflammation in the body that accelerates aging) *via* several mechanisms, including mitochondrial biogenesis and mitophagy through the PINK1/Parkin pathway (Zhao et al., 2021). It has recently been discovered that exercise, by increasing NAD^+^ levels, helps improve the function of SIRT1, thereby enhancing PINK1/Parkin mitophagy (Zhao et al., 2021).

#### Chemical Compounds

A 2017 *in vitro* study found that the neuroprotective role of resveratrol in AD results from its ability to induce mitophagy *via* upregulation of Parkin (Wang et al., 2018). In this study, cells treated with Aβ also showed an increase in Parkin compared with the control. In a recent study by Gao et al., tetrahydroxy stilbene glucoside (TSG) from the plant *Polygonum multiflorum* was shown to have neuroprotective effects *via* reducing the inflammatory cytokines and enhancing the Parkin-mediated mitophagy. Treating defective microglia with TSG upregulated both Parkin and PINK1 and, in higher doses, Beclin-1 as well. Out of both PINK1 and Parkin knockdown cells treated with TSG, those lacking PINK1 responded substantially less to TSG autophagy promotion. Moreover, this reduction was more than the case with Parkin knockdown cells, suggesting that TSG promoted PINK1 actions both directly and indirectly more than Parkin (Gao et al., 2020). However, higher levels of TSG decreased Parkin and PINK1 levels significantly (Gao et al., 2020), suggesting that excessive mitophagy may result in self-regulation. In another study, human neural cells treated with the mitophagy-inducing compound urolithin A showed higher amounts of parkin, PINK1, and Beclin-1. *In vivo* administration of this compound, along with another compound, actinonin, in APP/PS1 mice increased PINK-1(Fang et al., 2019). The interesting part was that urolithin A could inhibit Tau phosphorylation, and this action was interrupted in PINK1-knockdown cells, suggesting that this mitophagy could inhibit Tau phosphorylation (Fang et al., 2019). Moreover, both urolithin A and actinonin could prevent Tau phosphorylation *via* PINK1-dependent mitophagy (Fang et al., 2019). Finally, a study on an *in vitro* model of AD reported that valinomycin increased PINK1/Parkin mitophagy, enhanced Parkin translocation, and significantly reduced the number of mitochondria, as well as the Aβ level (Xiong et al., 2020). In this study, again, enhancement of PINK1 was higher than Parkin in the treated cells (relative significant increase vs. relative insignificant increase, respectively) (Xiong et al., 2020). The interesting finding in this study was that increasing valinomycin treatment time from 3 to 6 h increased the PINK1 and Parkin levels, while increasing the time from 6 to 12 h decreased them.

In a recent *in vivo* study, β-Asarone, a neuroprotective agent and the main component of *Acorus tatarinowii*, increased the expression of PINK1, Parkin, and Beclin-1 (Han et al., 2020). However, contrary to the above studies, lower levels of Parkin were found in the brains of AD models compared with control. Another study revealed that treatment of cells with Aβ downregulated the levels of Parkin and PINK1, while their studied compound, 6”‘-Feruloylspinosin, could prevent the detrimental effects of Aβ on Parkin and PINK1 overexpression (Yang et al., 2020). Relative expression of PINK1 was more enhanced with the compound (reached an insignificant difference with the control) than that of Parkin (difference with the control remained still significant), suggesting a higher effect on PINK than Parkin (Yang et al., 2020).

Melatonin was also shown to have neuroprotective effects *via* enhancement of mitophagy in APP/PS1 mice. However, contrary to the rest of the similar studies in this area, they observed a decrease in Parkin, as a sign of mitochondrial health; and the mice who received melatonin had better performances in memory tests and reduced levels of Parkin compared with AD controls (Sun et al., 2020).

Normally, it is believed that an agent or act, which increases the expression of Parkin, triggers mitophagy and helps restore mitochondrial homeostasis. What underlies this conflict cannot be fully understood without experiments; however, it might be the case of time, as once discussed in the previous section: elevation of Parkin at early stages of the disease in order to defend and protect, but failing the detrimental multidimensional effects of Aβ and hTau. Here, again, at early stages of compound administration (or exercise, respectively), the Parkin expression level increases, restoring mitophagy. Then, as the compound effects progress, dysfunctional mitochondria decrease in a load and quantity, allowing for Parkin levels back to normal (Gao et al., 2020; Xiong et al., 2020). The determining factor here is the time when assessments were done, especially that this study designed a long-term treatment, and treatment days were maximum among other similar studies. However, as melatonin is not a complete cure to AD, this time effect cannot be fully accepted, as the damage will never be totally removed (the decrease cannot reach the normal states).

These inconsistencies between the results of the studies cause controversies over analyzing Parkin level changes. Another factor might be that whether the Parkin level was assessed in only mitochondria or the whole cell. Except for one study, all other studies used whole cell or tissue for protein level assessment, and the controversies were within these studies (Table 1), ruling out the effects of Parkin assessment variations.

Finally, memantine is a well-known drug used in AD. In addition to its other roles against this disease, it appears that this drug helps restore mitophagy in both neurons lacking the Parkin gene and healthy controls, indicating a Parkin-independent pathway (Hirano et al., 2019).

**Figure 4:**
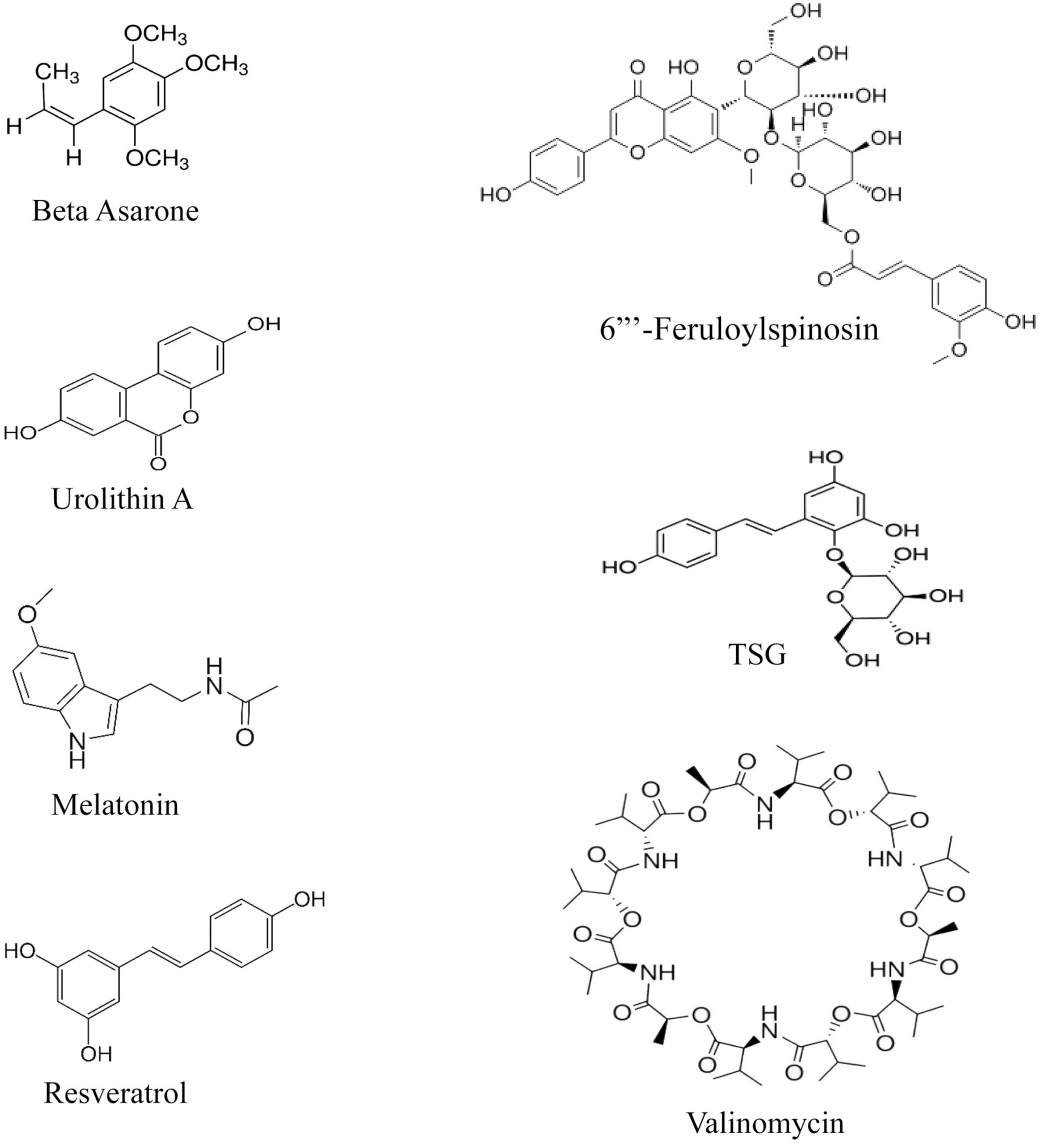
Chemical structures of the enhancers of the PINK/Parkin pathway.

Having a glance at the chemical structure of these compounds, it is implied that most of the structures follow a pattern of 3-Phenylethylene or its bioisosteres (Figure 4). Nevertheless, it is clear that data on their chemical-structure properties still extremely lack. The provided compounds are all either selected based on traditional medicine suggestions or have shown enhancing effects on autophagy in general, e.g., resveratrol was shown to activate SIRT1 in human umbilical vein endothelial cells (Guo et al., 2013). Since PINK1 is required to initiate the cascade on one hand, and these studies report increased PINK1 and Parkin by these agents on the other, it would be suggestable that increase in PINK1 and Parkin would compensate the lacks of the proteins in a brain undergoing rapid inflammation and oxidative stress. What happens in the cases of patients with neurodegeneration is innate insufficiency of these proteins, which cannot compete rapid aging of the brain, aside from deteriorating effects of amyloid beta and Tau on Parkin. It is, therefore, hypothesized that the effective agents increase PINK1 and Parkin to assist initiation of mitophagy. The exception is valinomycin, which has a structure close to aminoglycosides and supposedly exerts effects by depolarizing mitochondrial membrane, a supplementary effect to increase the PINK1 and Parkin levels (Rakovic et al., 2019). However, we are not aware of the exact site of action of these compounds that influences the Parkin pathway, nor have we yet found a compound that specifically interacts with this pathway and not the rest, since neuroactive compounds usually interact with many active sites in the brain. Moreover, the similarity of 3-phenylethylene with other neuroactive compounds, such as dopamine, indicates that development of a drug based on this SAR (structure activity relationship) may not be feasible, since many adverse reactions should be avoided, considering the fact that the origin of these compounds, which were, therefore, assumed to have effects on the Parkin pathway, was experimental, either through traditional medicine or recent discoveries about neuroprotective effects.

### Deubiquitination, a Checkpoint to Parkin-Mediated Mitophagy

Since Parkin is a ubiquitin, and since mitophagy is a programmed pathway in the cell, deubiquitinating enzymes [ubiquitin-specific peptidases (USPs)] play as the checkpoints to mitophagy. This is done to prevent excess mitophagy under stress conditions. An *in vitro* study by Wang et al. has investigated the role of USP30 and 35 in regulating Parkin-mediated mitophagy (Wang Y. et al., 2015). The mitofusin protein 2 (MFN2), from a class of proteins responsible for mitochondrial content exchange, is thought to send signals for Parkin translocation. This translocation is the most known sign for the initiation of Parkin-mediated mitophagy. The translocase of outer mitochondrial membrane 20 (TOMM20) is the other molecule responsible for Parkin translocation, which acts in association with PINK1. The USP30 is hypothesized to regulate the levels of these proteins (Wang Y. et al., 2015). It is further reported that this protein has a role in the protection of other OMM from ubiquitination. Finally, upon chemical mitophagy induction, this protein delayed translocation of Parkin to the OMM, which is a consequence of MFN2 prevention, they hypothesize (Wang Y. et al., 2015).

The USP 35, however, exerts its regulatory effects through unknown mechanisms. However, as the protein dissociates from the mitochondria at the time of mitochondrial depolarization (suggestive of mitophagy time), the authors suggest that this molecule routinely regulates MFN2 activity, preventing Parkin translocation (Wang Y. et al., 2015).

Other USP molecules are found to have a role in deubiquitination; however, data on their detailed mechanisms in mitophagy and Parkinson's and Alzheimer's disease seem to be extremely insufficient. Although the USPs perform several roles in the body, targeting one of such pathways could potentially help enhance the Parkin mitophagy *via* removing the regulation.

### Future Prospects

The effects of Aβ and hTau on Parkin regulation seem yet to have controversies and remain to be further explored. Moreover, data on the role of mitophagy in microglia activation and enhancement in AD models seem to be extremely limited. Since both Aβ/hTau and Parkin have contradictory roles against each other, their roles should be studied in detail.

Considering the probable role of SIRT 3, 4, and 5 proteins in mitophagy, it is recommended that future studies on neurodegeneration focus on the correlation between mitochondrial sirtuins and the Parkin pathway.

Almost all the non-clinical studies did not include gender differences in memory retention, although this is an interfering factor.

It appears that neuroprotective treatments targeting Parkin-mediated mitophagy are found with an increase in Parkin, which, again, requires exploration. The certain result could add a dimension in the evaluation of future neuroprotective agents.

## Conclusion

Studies on Parkin-mediated mitophagy seem to provide a promising future in ameliorating neurodegeneration and cognitive decline in AD (Moloudizargari et al., 2017). However, there is a considerable lack of experimental data on this area. It seems that, in molecular studies, an increase in Parkin is the result of damage to mitochondria and is observed in AD compared with healthy controls. However, by the time Aβ and hTau overcome compensatory systems of body, including Parkin's, the level significantly drops. This should be the case of what is observed at the clinical level. Certainty in this area needs further investigations. Moreover, it seems that a proper range/optimum dose exists for Parkin-enhancer compounds since higher exposures have yielded opposite results in some studies.

In summary, it appears that Parkin is a multifunctional protein preventing pathogenesis of AD, whose interactions with AD proteins are not yet fully understood. Discovering the gaps is strongly encouraged since it would help with a more profound and earlier diagnosis of Alzheimer's disease.

## Data Availability Statement

The original contributions presented in the study are included in the article/supplementary material, further inquiries can be directed to the corresponding author/s.

## Author Contributions

SG: idea suggestion, final screen, data extraction, writing of the manuscript, and extracting figures and tables. HH-A: systematic search and screen, sketching the graphs, and revision of the manuscript. MA: supervision. AH: systematic search and screen. All authors contributed to the article and approved the submitted version.

## Conflict of Interest

The authors declare that the research was conducted in the absence of any commercial or financial relationships that could be construed as a potential conflict of interest.

## Publisher's Note

All claims expressed in this article are solely those of the authors and do not necessarily represent those of their affiliated organizations, or those of the publisher, the editors and the reviewers. Any product that may be evaluated in this article, or claim that may be made by its manufacturer, is not guaranteed or endorsed by the publisher.
